# Special Issue “Microbial Endophytes: Functional Biology and Applications”: Editorial

**DOI:** 10.3390/microorganisms11040918

**Published:** 2023-04-01

**Authors:** Ajay Kumar, Gustavo Santoyo, James F. White, Virendra Kumar Mishra

**Affiliations:** 1 Centre of Advanced Study in Botany, Banaras Hindu University, Varanasi 221005, India; 2 Instituto de Investigaciones Químico Biológicas, Universidad Michoacana de San Nicolás de Hidalgo, Morelia 58030, Mexico; 3 Department of Plant Biology, Rutgers University, New Brunswick, NJ 08901, USA; 4 Institute of Environment and Sustainable Development, Banaras Hindu University, Varanasi 221005, India

Plants harbour various microbial communities, including bacteria, fungi, actinomycetes, and nematodes, inside or outside their tissues. These microbial communities play a significant role in growth promotion and provide protection from pathogen invasion and different biotic and abiotic stresses [1]. The microbes that reside in the plant tissues without apparent signs of infections are termed endophytes [2,3]. Technological advancements have allowed us to determine that each plant has at least some endophytes in its life cycle [4,5]. The endophytes within the tissue share complex interactions and regulate the metabolic activities of the host plants, which are required for optimum growth, development and protection from stresses [5]. In general, the microbial endophytes commonly interact with the host via mutualism, commensalism and opportunism. In the mutualistic relation, the endophytes and the host plant benefit each other. Microbes fulfil the nutritional requirements and modulate phytohormones; in return, the plant provides the microbes with a home and food [4].

The better colonization efficacy and acclimatizing potential against abiotic stress such as salinity and drought make the endophyte the most promising microbial entity compared to other microorganisms [6]. In the recent past, endophytic microbes, especially bacteria and fungi, have frequently been utilized as microbial inoculants to enhance agricultural productivity via various mechanisms such as phytohormone modulation, phosphate solubilization, and nutrient acquisition [5,7]. In addition, the secretion of bioactive compounds, antibiotics and siderophore products makes them suitable biocontrol agents [8]. However, very few percentages of endophytic strains have been characterized and commercialized in the wake of such technological advancements. Therefore, the functional biology and hidden potential of endophytic microorganisms must be explored.

The Special Issue “Microbial Endophytes: Functional Biology and Applications” aims to compile the latest research on endophytes, their significant contribution to sustainable agriculture as biofertilizers and biocontrol agents and their role in abiotic stress management and pharmaceutical industries, as well as in the synthesis of bioactive compounds. This Special Issue contains six research papers and two review papers. The article led by Semenzato et al. [9] analyses how the endophytic bacteria modulate the synthesis of bioactive compounds and their antagonistic behaviour against opportunistic pathogens. The authors briefly studied the genome sequence of endophytic strains *Metabacillus dongyingensis Priestia megaterium Paenibacillus xylanexedens Priestia megaterium* isolated from the *Origanum vulgare* L. Further in silico analysis identified the gene cluster responsible for antimicrobial activity. In another study, Singh et al. [10] isolated eight endophytic bacterial strains from the *Momordica charantia* L. root. All the strains showed plant growth promotion ability and variably utilized carbon and nitrogen resources as substrates. In addition, some strains, such as R1 and RS6, showed salinity tolerance of up to 10%, and most of the strains were resistant to different antibiotics. The isolated strains can be used as biofertilizers and for abiotic stress management.

Ntana et al. [11] detailed the ways in which the secreted metabolites modulate the specific interaction between the endophytic strain *Serendipita indicia and Solanum lycopersicum* and other microbes present in the niche. In brief, the sample plant *Solanum lycopersicum* was inoculated with the spores of *S. indica*. Further, their inoculation effect was observed by analysing the gene expression pattern of leaves and roots of endophyte-inoculated plants through RNAseq analysis. The endophyte inoculation strongly induces the gene responsible for synthesizing polyacetylenes and some specific terpenes. Further, another study by Roodi et al. [12] reported two endophytic fungi *Beauveria bassiana* and *Pseudogymnoascus pannorum*, which were inoculated into three different species of *Brassica*, namely *Brassica napus*, *Br. rapa* and *Br. Oleracea*. The inoculation of the endophytic fungal strains showed mutualistic behaviour with the host plant. It significantly inhibited the growth of the pathogen *Leptosphaeria maculans* causal agent of phoma stem canker in *Brassica.*

In another published article, Chen et al. [13] evaluated the inoculation effect of *Epichloë* endophyte in breaking dormancy of *Achnatherum inebrians* seeds. In the study, the authors revealed that inoculation of endophytes significantly breaks the dormancy of *A. inebrians* seeds and enhances their rate of germination. In addition, it also enhanced the total soluble sugar and phytohormones such as indole-3-acetic acid and gibberellin in the treated seeds. In another published article, Tufail et al. [14] reported a global meta-analysis of the effects of endophyte inoculation on the morphological and physiological parameters of plants. The analysis revealed that inoculation of bacterial endophytes significantly enhanced the morphology, such as root and shoot length, number of leaves, antioxidative enzymes and chlorophyll contents of the plants. It also maintained the ionic balance under saline conditions. Furthermore, authors also observed that endophytes inoculation significantly enhanced the growth and development of plants and mitigated the challenges of salinity stress in salt-sensitive and salt-tolerant plants. However, the higher efficacy of endophyte inoculation has been observed in the plants growing under salinity stress conditions.

Fernando et al. [15] briefly explored the interaction of the endophytic strain *Epichloë* with the Pooidae grasses. They analysed the metabolic potential of host–endophyte interactions and the crucial role of *Epichloë* in biotic stress management. This study shows that *Epichloë* can be used as a natural biocontrol agent against different phytopathogens. Finally, Verma et al. [16] reviewed the impact of salinity or drought stress on crop plants and their mitigation strategies using endophytic microbial strains. The authors briefly reported how abiotic stress, including draught or salinity, elevates the plant’s immune response and releases reactive oxygen species. The generation of reactive oxygen severally affects the morphological and biochemical aspect of plants, which ultimately leads to death. The inoculation of endophytic strains modulates the phytohormones, quenches reactive oxygen, protects the plants from abiotic stress and modulates the growth and development of plants.

In conclusion, this Special Issue compiles works related to fundamental aspects of microbial endophytes and their beneficial interactions with their plant hosts, providing various services. There is no doubt that future inoculants will contain microbial endophytic agents that will benefit the growth and production of various crops, contributing to world food security and preserving a healthy agro-environment.

## Data Availability

Not applicable.
